# Bioinformatic prediction of putative conveyers of O-GlcNAc transferase intellectual disability

**DOI:** 10.1016/j.jbc.2022.102276

**Published:** 2022-07-19

**Authors:** Conor W. Mitchell, Ignacy Czajewski, Daan M.F. van Aalten

**Affiliations:** 1 Department of Molecular Biology and Genetics, Aarhus University, Aarhus, Denmark; 2 Division of Cell and Developmental Biology, School of Life Sciences, University of Dundee, Dundee, United Kingdom

**Keywords:** O-GlcNAc, neurodevelopment, intellectual disability, bioinformatics, glycobiology, cell signaling, gene expression, AHCY, adenosyl homocysteinase, CDG, congenital disorder of glycosylation, CTE, C-terminal extension, DD, developmental delay, EESC, embryonic stem cell, GO, gene ontology, ID, intellectual disability, mESC, mouse embryonic stem cell, OGT, O-GlcNAc transferase, PDB, Protein Data Bank, PTM, posttranslational modification, TPR, tetratricopeptide

## Abstract

Protein O-GlcNAcylation is a dynamic posttranslational modification that is catalyzed by the enzyme O-GlcNAc transferase (OGT) and is essential for neurodevelopment and postnatal neuronal function. Missense mutations in OGT segregate with a novel X-linked intellectual disability syndrome, the OGT congenital disorder of glycosylation (OGT-CDG). One hypothesis for the etiology of OGT-CDG is that loss of OGT activity leads to hypo-O-GlcNAcylation of as yet unidentified, specific neuronal proteins, affecting essential embryonic, and postnatal neurodevelopmental processes; however, the identity of these O-GlcNAcylated proteins is not known. Here, we used bioinformatic techniques to integrate sequence conservation, structural data, clinical data, and the available literature to identify 22 candidate proteins that convey OGT-CDG. We found using gene ontology and PANTHER database data that these candidate proteins are involved in diverse processes including Ras/MAPK signaling, translational repression, cytoskeletal dynamics, and chromatin remodeling. We also identify pathogenic missense variants at O-GlcNAcylation sites that segregate with intellectual disability. This work establishes a preliminary platform for the mechanistic dissection of the links between protein O-GlcNAcylation and neurodevelopment in OGT-CDG.

Pathogenic missense variants in O-GlcNAc transferase (OGT) give rise to OGT congenital disorder of glycosylation (OGT-CDG), a rare, clinically heterogenous intellectual disability (ID) syndrome where the underlying molecular etiology is unknown. By filtering the O-GlcNAc proteome for putatively functional O-GlcNAc sites and O-GlcNAc sites mutated in ID patients, we have identified a subset of the O-GlcNAcome, the hypo-O-GlcNAcylation of which may give rise to OGT-CDG.

O-GlcNAcylation, the covalent attachment of monosaccharide units of β-linked GlcNAc to the hydroxyl groups of protein serine/threonine (Ser/Thr) residues, is a dynamic and highly conserved posttranslational modification (PTM) occurring on thousands of nucleocytoplasmic and mitochondrial proteins (1). This dynamic PTM is catalyzed by the OGT (2, 3). OGT is a type B glycosyltransferase, composed of 13.5 N-terminal tetratricopeptide (TPR) repeats and a C-terminal glycosyltransferase domain (4). The N-terminal TPRs confer both substrate specificity and serve as a molecular hub for the assembly of multiprotein complexes (5). The C-terminal glycosyltransferase domain possesses both sugar transfer activity and a noncanonical protease activity, the latter being required for proteolytic maturation of the cell cycle regulator HCF-1 (6). The antagonistic enzyme to OGT, O-GlcNAc hydrolase, removes the O-GlcNAc modification (7).

OGT is most abundant in the brain (8), where O-GlcNAc mass spectrometric studies have identified thousands of O-GlcNAc-modified proteins involved in synaptic plasticity, excitatory neurotransmission, and neuronal activity–dependent gene transcription (9, 10, 11). However, the site-specific roles of O-GlcNAc in regulating neurodevelopmental and postnatal neuronal processes remains poorly understood, with the functional consequences of O-GlcNAcylation understood for only a handful of neuronal OGT substrates. Despite this, previous work has established the role of O-GlcNAc in modulating dozens of neurodevelopmental processes, including axonal growth (12), dendritic spine maturation (13), and synaptic vesicle dynamics (14). Furthermore, hippocampal-specific depletion of OGT abrogates long-term potentiation and excitatory synapse maturation, key processes underpinning learning and memory, potentially due to disrupted α-amino-3-hydroxy-5-methyl-4-isoxazolepropionic acid receptor trafficking (13). Additionally, O-GlcNAcylation is essential for dopaminergic neuron survival, with loss of OGT activity leading to massive apoptosis and near-abolition of dopamine release as a second order consequence (15). At the organismal level, O-GlcNAcylation is protective against neurodegeneration and age-related decline in learning and memory (16). Strikingly, pan-neuronal KO of OGT results in perinatal lethality in mice, which is immediately preceded by locomotor dysfunction and hyperphosphorylation of the Alzheimer’s disease associated protein Tau (17). Taken together, O-GlcNAcylation is an essential PTM for neurodevelopment and neuronal function, though a comprehensive mechanistic understanding of how O-GlcNAc functions in the brain and developing central nervous system is lacking.

Recently, several groups have independently reported missense variants in the *OGT* gene that segregate with ID (18, 19, 20, 21). IDs are a group of clinically and genetically heterogenous neurodevelopmental disorders, collectively characterized by an intelligence quotient of less than 70, as well as compromised adaptive and social behavior (22). ID can result from mutations in one of over 700 genes and typically co-occurs with failure to reach key milestones in social, motor, or language development (known as developmental delay) (22). The ID syndrome which segregates with *OGT* missense variants, recently named the OGT-CDG, is symptomatically highly heterogenous (23). Patients typically present with ID, developmental delay, hypotonia, hearing/visual impairments, facial dysmorphia, long-thin fingers, clinodactyly, short stature, and eye abnormalities (23). However, additional conditions such as epilepsy and microcephaly are comorbid with OGT-CDG. This clinical heterogeneity strongly suggests that multiple signaling cascades and developmental processes are disrupted in OGT-CDG, further complicating therapeutic strategies and dissection of molecular etiology. Moreover, exactly how missense mutations in *OGT* give rise to OGT-CDG is not clear. Pathogenic OGT mutations have been reported in both the substrate specificity conferring TPRs (20, 21) and the glycosyltransferase domain, leading to divergent hypotheses regarding molecular etiology. For example, it has been hypothesized that OGT missense variants abrogate proteolytic maturation of HCF1, preventing normal cell cycle progression (23). However, OGT modifies >4000 substrates, and reduced OGT activity toward a single substrate cannot explain the clinical heterogeneity observed in OGT-CDG patients. Alternatively, it has been hypothesized that the missense variants reduce OGT stability, indirectly leading to reduced protein O-GlcNAcylation. While this has been observed for the OGT^L254F^ variant, where the TPR domain displays reduced thermal stability *in vitro* (24), this has not been reported for other OGT-CDG mutations. The majority of reported OGT missense variants map to the TPRs (20, 21, 23), leading to the hypothesis that selective loss of OGT interactors may give rise to OGT-CDG. However, no data have surfaced to date which substantiates this hypothesis. Instead, characterization of the OGT-CDG N648Y and N567K variants revealed that mutations in the OGT glycosyltransferase domain lead to lower global O-GlcNAcylation levels in mouse embryonic stem cells (mESCs) and *Drosophila melanogaster*, impair proteolytic maturation of HCF1, and strongly attenuate O-GlcNAcylation of the physiological OGT substrate Table 1 *in vitro* (18, 19). Collectively, these data indicate that hypo-O-GlcNAcylation of OGT substrates at particular developmental time points, due to catalytic deficiency of OGT, may give rise to OGT-CDG. The identity of these OGT substrates, referred to in this manuscript as “phenotypic conveyers,” is unknown. Identification of these phenotypic conveyers would allow elucidation of the molecular etiology of OGT-CDG and further develop an understanding of the roles of O-GlcNAcylation in neurodevelopment and neuronal function through dissection of the site-specific roles of O-GlcNAc on these proteins.

**Table 1:** Missense variants at conserved sites of O-GlcNAcylation identified in patients with ID/DD

Gene	Variant	Patient condition	Pathogenicity	PolyPhen score
*PUM1*	S802F	Spinocerebellar ataxia 47	Uncertain significance	0.921
*TUBB2B*	S172P	Cortical dysplasia, complex, with other brain malformations	Pathogenic	
*TUBB2B*	S172L	Cortical dysplasia, complex, with other brain malformations	Likely pathogenic	
*DOCK7*	S190N	Epileptic encephalopathy, early infantile, 23	Uncertain significance	0.995
*DOCK7*	S190G	Epileptic encephalopathy, early infantile, 23	Uncertain significance	0.989
*ATRX*	S594C	Alpha thalassemia-X-linked intellectual disability	Uncertain significance	0.993
*HCF1*	T556M	Mental retardation 3, X-linked	Uncertain significance	0.959
*KMT2A*	S1858I	Wiedemann–Steiner syndrome	Uncertain significance	0.855

The ClinVar database was screened for pathogenic missense mutations at O-GlcNAc sites reported in a recently published O-GlcNAc database (see (46)). Patient condition and the associated pathogenicity of the missense mutation are summarized. Where the significance of the missense mutation is uncertain, PolyPhen HumVar scores are provided, where default cut-offs of 0.15 for “likely benign” and 0.85 for “probably damaging” are applied.

One of the challenges in identifying phenotypic conveyers of the OGT-CDG phenotype is the lack of depth of information provided by O-GlcNAc site-mapping studies. O-GlcNAc proteomic studies have expanded the size of the O-GlcNAcome dramatically in the past 15 years (9, 10, 11, 25, 26). However, these same studies typically identify O-GlcNAc sites without subsequently predicting or attempting to ascribe functions to identified O-GlcNAc sites. This is crucial, as the presence of O-GlcNAc on a protein does not necessarily imply a functional consequence. For example, of the 11 O-GlcNAc sites identified on Keap1, only one—S104—modulates downstream NRF2 signaling in response to stress (27). Similarly, the O-GlcNAcylation stoichiometry of the methionine cycle regulator adenosyl homocysteinase (AHCY) is predominantly contributed by a single O-GlcNAc site, whereas the remaining three sites make negligible contributions to AHCY O-GlcNAcylation stoichiometry and lack an identifiable function (28). Consequently, although the O-GlcNAcome stands at over 4000 proteins, it is difficult to predict if and how hypo-O-GlcNAcylation of specific O-GlcNAcylated neuronal proteins would alter protein function and subsequently contribute to the OGT-CDG phenotypes.

In this study, a filter-based bioinformatics approach was used to predict the function of O-GlcNAc sites on neuronal proteins that have already been reported to be linked to ID. Utilizing O-GlcNAc site-mapping data in combination with sequence conservation, structural data, clinical databases, and literature review, we predict the existence of 38 functional O-GlcNAc sites across 22 neuronal proteins. In most cases, the role of the identified O-GlcNAc site is unknown. As these proteins are pathogenically mutated in reported ID/developmental delay (DD) syndromes and harbor putatively functional O-GlcNAc sites, their hypo-O-GlcNAcylation may contribute to OGT-CDG etiology. Additionally, by screening the ClinVar database against a recently compiled O-GlcNAc site database, we identify eight missense variants at O-GlcNAcylation sites in ID/DD patients, further suggesting a convergence of the O-GlcNAc proteome and ID etiology.

## Results and discussion

### A filter-based approach identifies OGT-CDG candidate conveyers

**Figure 1:**
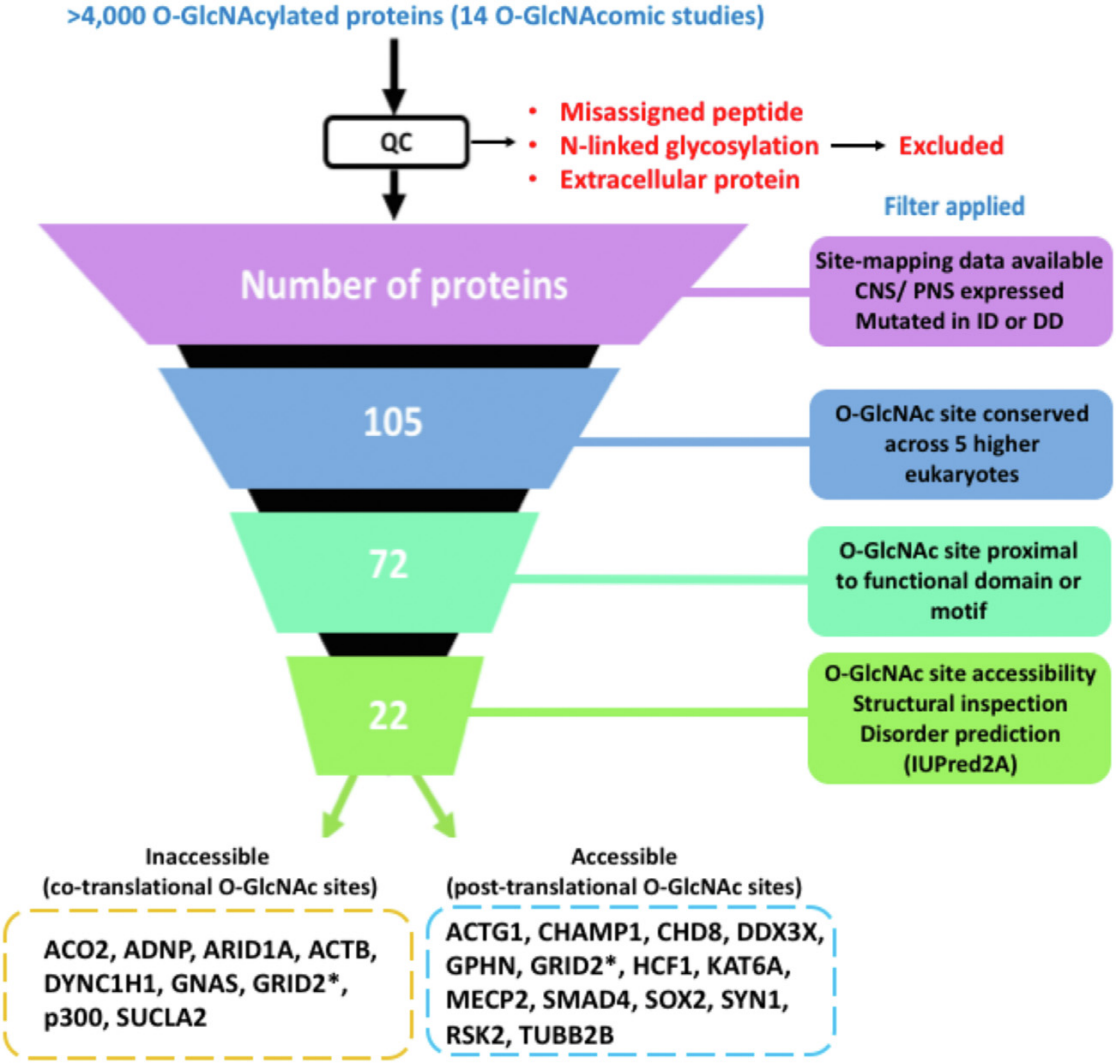
**Illustration of the rational, filter-based approach for identification of candidate conveyers of the OGT-CDG phenotypes.** Site-mapping data for O-GlcNAcylated proteins identified in 14 O-GlcNAc mass spectrometric (O-GlcNAcomic) studies of mouse, rat, and human CNS tissue samples and cell lines were pooled and applied to the above illustrated, filter-based approach (see Experimental procedures). The relevant filters applied at each level are annotated. GRID2 (∗) was predicted to harbor both cotranslational and posttranslational O-GlcNAc sites on the basis of IUpred disorder prediction. CDG, congenital disorder of glycosylation; CNS, central nervous system; OGT, O-GlcNAc transferase.

To identify candidate conveyers of the OGT-CDG phenotypes, we used a filter-based approach integrating O-GlcNAc site-mapping data, available structural data, clinical databases, and the literature. O-GlcNAc site-mapping data were pooled manually from 14 studies that reported the O-GlcNAcomes of various cell lines (HEK293 (25), mESCs (29), HeLa (30), T-lymphocytes (26)) and tissue extracts (rat brain (31), mouse brain (32), and mouse synaptosomes (9, 11, 33, 34)). Next, we extracted O-GlcNAcylated proteins, where missense mutations in the corresponding coding gene were reported to give rise to ID/DD, using the list of ID/DD-associated genes compiled by Vissers *et al.* (22) and the DECIPHER database (35) as reference points. The DECIPHER database reports on the pathogenicity and associated phenotype of variants in a given gene, and proteins passed this filter if their corresponding gene was the sole pathogenic variant in a patient with ID or DD. About 105 O-GlcNAcylated proteins, segregating with X-linked ID passed this first filter (see File S1 for list of O-GlcNAcylated X-linked ID proteins). Sequence conservation of O-GlcNAc sites was assessed from sequence alignments of higher eukaryotic orthologues (human, mouse, rat, rabbit, and sheep), leading to rejection of candidates where the O-GlcNAcylated Ser/Thr was poorly conserved (absent in ≥1 species). Subsequently, the available literature was used to investigate whether such conserved O-GlcNAc sites resided in functionally important domains or motifs of the protein. Examples of such functionally important domains or motifs include protein:protein interaction domains, protein:nucleic acid interaction domains, enzymatic active sites, or metal-binding sites. These filters reduced the number OGT-CDG candidate conveyers in the whole O-GlcNAcome to 22 candidate proteins (Fig. 1).

We further segregated O-GlcNAc sites on our 22 candidate proteins into two groups. Crystallographic studies of OGT in complex with substrate peptides have suggested that the active site and TPR domain of OGT accommodate disordered regions of proteins adopting an extended conformation (36, 37, 38)^,^. However, several examples exist of O-GlcNAc sites residing in ordered regions of the protein fold and these studies do not yet explain why (11, 28, 36). Importantly, Ser/Thr residues in ordered secondary structures would be inaccessible to the OGT active site. An example of this is AHCY, which is activated by O-GlcNAcylation at Thr136, despite this site residing in an α-helix that is theoretically inaccessible to the OGT active site (28). Recently, it has been discovered that O-GlcNAcylation can also occur cotranslationally on disordered peptidyl-tRNAs, as they emerge from the ribosome (39). It is possible that O-GlcNAc sites in inaccessible, ordered regions of the protein fold represent a subpopulation of O-GlcNAc sites that are cotranslationally modified by OGT. We therefore further segregated the list of candidate proteins/sites into those with accessible (in surface-exposed, disordered protein regions) and inaccessible (within sequestered, ordered regions of the protein fold) O-GlcNAc sites. To achieve this, we used available Protein Data Bank (PDB) structures of proteins or where such data were unavailable, the disorder predictor IUpred2A (40). Overall, our filter-based approach (illustrated in Fig. 1) suggests that of the 22 candidate conveyors of the OGT-CDG phenotypes, 13 are posttranslationally O-GlcNAcylated, eight cotranslationally O-GlcNAcylated, and one candidate (GRID2) harbors both cotranslational and posttranslational O-GlcNAc sites. For summaries of each OGT-CDG candidate and details regarding conserved O-GlcNAc site(s) of interest, see Table S1.

**Figure 2:**
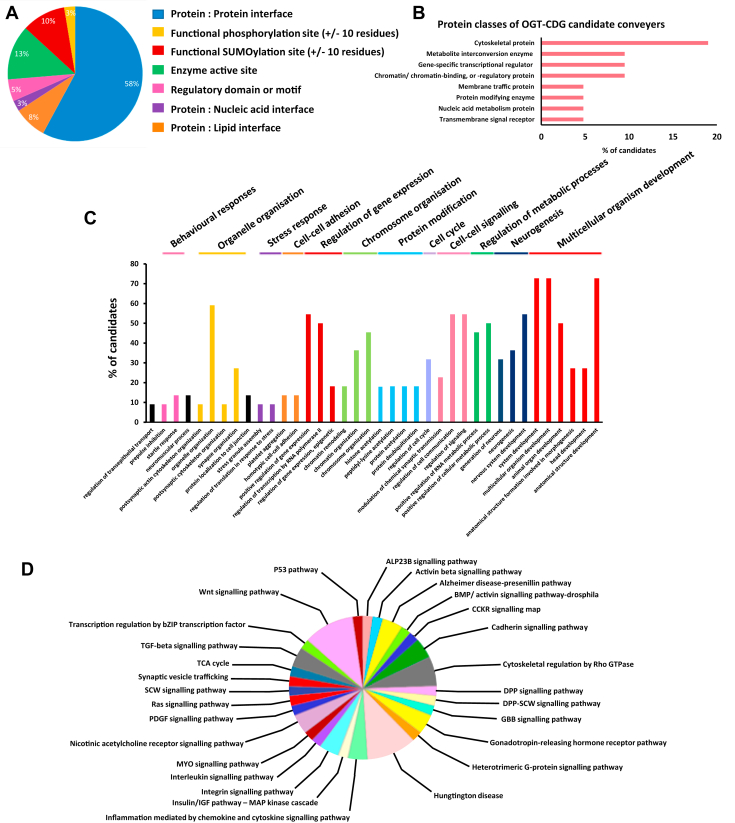
**Classification of OGT-CDG candidate conveyers by protein class, positioning of O-GlcNAc site(s), and convergent signaling pathways and biological processes.***A*, percentage of O-GlcNAc sites on OGT-CDG candidate conveyers, which were predicted to be functional based on either: (I) present on a protein:protein interface; (II) adjacent (±10 residues) from another PTM site, which has been validated as functional in previous work; (III) within or directly abutting against an enzyme active site; and (IV) present within a known regulatory region. *B*, stratification of OGT-CDG candidate conveyers by class of protein using PANTHER, UniProt accessions of human homologs were used (see File S1). *C*, classification of biological processes involving OGT-CDG candidate conveyers using the Gene Ontology (GO) resource. GO terms were clustered manually into common groups and highlighted. *D*, signaling pathways involving OGT-CDG candidate conveyers were extracted using PANTHER analysis of Gene IDs, which were manually retrieved from NCBI. CDG, congenital disorder of glycosylation; OGT, O-GlcNAc transferase.

Of the 39 candidate O-GlcNAc sites, the majority (∼58%) were predicted to be functional based on their localization to a protein:protein binding interface. Four candidate O-GlcNAc sites were either reported as being phosphorylated or SUMOylated or as proximal (±10 residues) to a functional phosphorylation/SUMOylation site (Fig. 2*A*). The remaining sites were either located in enzyme active sites (RSK-2, GNAS), a regulatory domain/motif (KAT6A, SMAD4) or a protein:nucleic acid interface (ADNP, ARID1A; Fig. 2*A*). Classification of OGT-CDG candidate conveyers by protein class, using PANTHER (Protein ANalysis THrough Evolutionary Relationships) (42), showed a substantial portion of candidates were cytoskeletal or microtubule-associated proteins (19%; TUBB2B; ACTB; ACTG1; DYNC1H1; Fig. 2*B*). Other classes of protein present in our candidate list were transcriptional regulators (SOX2, SMAD4), chromatin binding or regulatory proteins (P300, KAT6A), and metabolic enzymes (ACO2; SUCLA2). Transmembrane signaling receptors (GRID2), protein modifying enzymes (RSK-2), and nucleic acid metabolism enzymes (MECP2) were also identified (Fig. 2*B*). Seven candidates were not annotated as belonging to a single protein class. Overall, our OGT-CDG candidate conveyers represent a diverse pool of proteins.

In addition to conserved O-GlcNAc sites residing in functional domains or regions of ID/DD-associated proteins, we also investigated whether poorly conserved O-GlcNAc sites on ID/DD-associated proteins may reside in interesting regions of a given protein. Toward this end, we analyzed whether the 105 ID/DD-associated O-GlcNAc proteins identified after the first filter harbored poorly conserved O-GlcNAc sites (O-GlcNAc Ser/Thr not conserved in ≥ 1 species) in functional domains/regions of the protein. Four hits (NDUFS3, aprataxin, PDHA1, and ankyrin G) were identified due to O-GlcNAc sites either localizing to mitochondrial targeting sequences (PDHA1, NDUFS3), the interaction site for PARP-1 (aprataxin), or an isoform-specific domain associated with regulation of protein localization (ankyrin G). These lower confidence hits are summarized in Table S2.

We subsequently used gene ontology (GO) analysis to assess possible convergence of candidate conveyers on a small set of biological processes and signaling pathways. Most of the candidates were annotated as being involved broadly in development and neurogenesis (Fig. 2*C*). Intriguingly, we also observed that some of our candidate conveyers were annotated as forming part of the stress response (DDX3X, DYNC1H1; see discussion on DDX3X later), a process known to be regulated by O-GlcNAcylation (41). Additional biological processes included cell cycle regulation, chromatin organization, regulation of gene expression, and protein modification (acetylation/ubiquitination, Fig. 2*C*). For all these biological processes, previous work has strongly suggested a functional role for O-GlcNAcylation in their regulation (6, 27, 39, 41, 42, 43). We further carried out pathway analysis using PANTHER and found that the candidate conveyers were involved in a number of signaling pathways. Of particular interest, we noted that Dpp signaling, Wnt signaling, and synaptic vesicle trafficking were hits (Fig. 2*D*). In *D. melanogaster*, Dpp (BMP4 in vertebrates) signaling is negatively regulated at the level of the bone morphogenic protein (BMP) receptor Sax by O-GlcNAcylation (44), and the stability of Wnt signaling nodes CK2 and β-catenin are negatively and positively regulated by O-GlcNAc, respectively (45, 46). O-GlcNAcylation of Synapsin I, which is also identified as a candidate conveyer in this study (Table S1), regulates presynaptic vesicle dynamics and the size of the reserve pool of synaptic vesicles (14). Thus, the OGT-CDG candidate conveyers identified here are involved in diverse biological processes and signaling pathways, many of which are already known to be regulated by O-GlcNAcylation. Future work, characterizing the function of O-GlcNAc on these candidate proteins, guided by the hypotheses we have presented, may reveal additional levels of O-GlcNAc-dependent regulation in these processes. Later, we discuss selected examples of candidate conveyers and their associated O-GlcNAc sites of interest.

### O-GlcNAcylation of cytoskeletal proteins suggests that dysregulated cytoskeletal dynamics may contribute to OGT-CDG pathology

Precise regulation of cytoskeletal dynamics is essential for the ability of initially spherical cell soma to give rise to distinct somatodendritic and axonal compartments (47). This process, termed neuronal polarization, is essential for the correct flow of electrochemical impulses and the trafficking of RNA, ribosomes, and ion channels to distal synapses (48). Indeed, disruption of cytoskeletal dynamics leads to neurodevelopmental disorders such as lissencephaly and cortical dysplasia (49). Regarding cytoskeletal proteins, our bioinformatic analysis predicted O-GlcNAc sites on both ß-tubulin (isoform TUBB2B) and actin (isoforms ACTB1 and ACTG1) to be important for regulating their respective proteins’ functions (see later).

**Figure 3:**
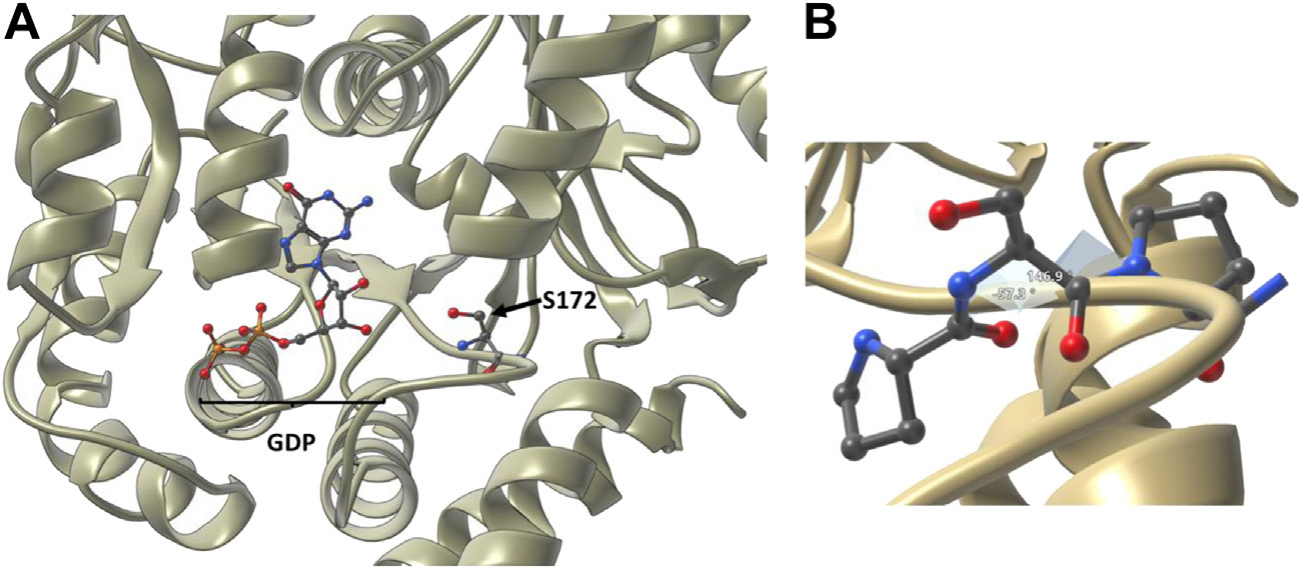
**The β-tubulin O-GlcNAc site S172 and associated backbone angles.***A*, crystal structure of β-tubulin from Lowe *et al*. (PDB entry: 1JFF (51) with S172 and bound GDP highlighted as ball and stick depictions. Note how the S172 hydroxyl points directly toward the bound GDP in the GTPase active site. *B*, measured dihedral angles for S172 (Phi = −57 °, Psi = 147 °). P171, S172, and P173 are highlighted as ball and stick depictions.

In our bioinformatic analysis, we identified S172 as a highly conserved ß-tubulin O-GlcNAc site. S172L and S172P mutations segregate with cortical dysplasia, complex, with other brain malformations, seven in several ID patients (49) (CDCBM7; See section “Identification of pathogenic missense variants at O-GlcNAcylation sites” and File S2). Additionally, S172 resides on a loop that forms part of the GTPase active site (Fig. 3*A*) (50, 51). Phosphorylation of S172 by CDK1, during mitosis, prevents GTP/GDP exchange, presumed to be due to hydrogen bonding interactions between the phosphate and GDP preventing GDP dissociation from the active site (50). The presence of a bulky GlcNAc moiety in the GTPase active site would likely sterically hinder GTP binding, thus reducing the pool of GTP-bound tubulin and inhibiting tubulin polymerization. Interestingly, Ji. *et al.*(52) previously observed that O-GlcNAcylated tubulin is exclusively monomeric, supporting the hypothesis that tubulin O-GlcNAcylation prevents microtubule assembly, though the functional sites of tubulin O-GlcNAcylation were not identified in this study. If S172 O-GlcNAcylation does regulate tubulin polymerization indirectly, through modulation of GTP binding affinity to tubulin, then hypo-O-GlcNAcylation of tubulin in the context of OGT-CDG could lead to increased rates of tubulin polymerization. This increased rate of tubulin polymerization, in-turn, could deplete the pool of monomeric tubulin, altering the structural plasticity of the microtubules in neurons and potentially preventing morphological changes in the dendrites and axons that are crucial for neuronal polarization (47). This hypothesis could be investigated by an *in vitro* polymerization assay, comparing TUBB2B^WT^ and TUBB2B^S172A^ polymerization rates (53).

In addition to the microtubule networks in neurons, the actin cytoskeleton plays an essential role in regulating neuronal polarization, and fine-tuning actin cytoskeleton dynamics and function severely impacts both axon differentiation (54) and growth cone motility (55) prior to synaptogenesis. Pharmacological inhibition of actin polymerization induces spontaneous axon differentiation from immature neurites (54). Furthermore, heterozygous actin loss-of-function variants have been reported in patients displaying ID, developmental delay, internal organ malformation, and growth retardation (56). The highly conserved actin O-GlcNAc site S365 lies on the interface between actin and its regulatory protein profilin (57). The S365 hydroxyl group points toward a E364-K126 salt bridge on the actin:profilin interface, with the S365 hydroxyl and E364 carboxylate only 3.3 Å apart (see PDB entry 2BTF (57)). A bulky O-GlcNAc moiety on S365 could therefore disrupt formation of this salt bridge to inhibit actin:profilin binding. Profilins stimulate the exchange of GDP for GTP on actin, thus regulating the size of the pool of polymerizable GTP-actin monomers (58). Knockdown of profilin IIa in mice leads to increased neurite outgrowth, ostensibly due to reduced actin polymerization and reduced F-actin density (59). Whether actin S365 O-GlcNAcylation inhibits actin polymerization through reduced affinity of actin for profilin and whether hypo-O-GlcNAcylation of actin at S365 alters actin polymerization in the context of OGT-CDG, will require further investigation.

In addition to S365, our bioinformatic analysis of the O-GlcNAcome identified S265 as a previously reported, conserved O-GlcNAc site on actin, which resides in a hydrophobic loop (residues 264–273). In the Holmes model of F-actin, this hydrophobic loop inserts into a pocket formed by an adjacent F-actin protomer in a manner analogous to a “socket” and a “plug,” with the loop constituting the plug and the socket formed by hydrophobic residues in the adjacent promoter (60). Holmes *et al.* (60) hypothesized that loss of this interaction between adjacent actin protomers would likely severely disrupt actin polymerization, as other electrostatic interactions along the length of F-actin may not be sufficient to compensate for loss of the hydrophobic interactions contributed by residues 264 to 273. The presence of O-GlcNAc on S265 therefore presents an interesting target for study and suggests that O-GlcNAc may regulate actin polymerization both directly (through disruption of actin protomer assembly) and indirectly (through reduced affinity for profilin).

### O-GlcNAcylation of DDX3X implicates dysregulated RNA metabolism in OGT-CDG etiology

**Figure 4:**
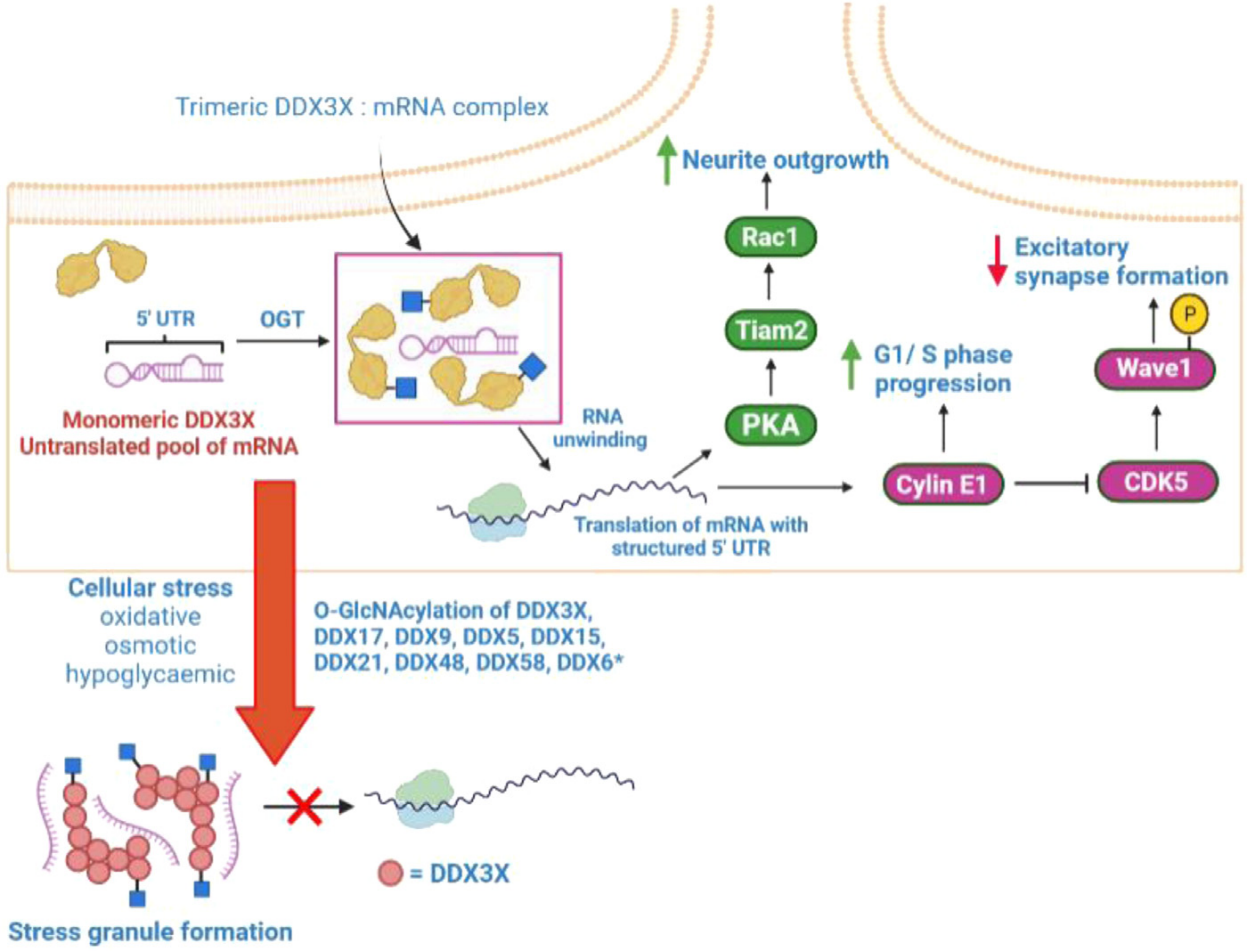
**The DDX3X O-GlcNAc sites S584 and S588, and hypothesized functions of DDX3X O-GlcNAcylation in RNA metabolism and neurodevelopment.** Illustrated summary of hypothesized function(s) of DDX3X O-GlcNAcylation. Briefly, DDX3X O-GlcNAcylation in the CTE at residues S584 and S588 may promote formation of functional DDX3X trimers. Functional DDX3X trimers subsequently promote the translation of signaling nodes in the Rac1/PKA and cyclin E1/CDK5 signaling axes. Additionally, in response to cellular stress, DDX3X and at least nine other DEAD-box RNA helicases (∗) may be O-GlcNAcylated as part of the stress response. CTE, C-terminal extension.

The filter approach identified a member of the DEAD box RNA helicase family—DEAD box RNA helicase 3 X-linked (DDX3X)—as a putative conveyer of the OGT-CDG phenotype. DDX3X is an ATP-dependent RNA helicase, which assembles into trimers capable of unwinding structured mRNA 5′ UTRs (61, 62). This local strand separation is essential for docking and scanning of mRNA for translation initiation sites, and DDX3X-mediated RNA unwinding is crucial for the translation of cell cycle progression genes (63) and components of the Rac1-PKA signaling axis required for neurite outgrowth (See Fig. 4) (64). Furthermore, DDX3X knockdown in bone marrow–derived macrophages reduces stress granule formation in response to oxidative stress by ∼50% (65). Stress granules are translationally repressive aggregates of ribonucleoproteins formed in response to cellular stresses such as glucose deprivation, osmotic stress, or oxidative stress to protect untranslated mRNAs from damage (66). Curiously, OGT knockdown in U2OS cells also abolishes stress granule formation following arsenite-induced osmotic stress (41), and mass spectrometric studies have identified at least nine DEAD box RNA helicase as being O-GlcNAcylated (26). However, exactly how OGT and stress granule formation are mechanistically linked is presently unknown (see Fig. 4 for illustrated hypothesis). Furthermore, missense mutations in the *ddx3x* gene are linked with a clinically heterogenous ID syndrome comprising of, though not limited to, developmental delay, hypotonia, corpus callosum hypoplasia, epilepsy, behavioral problems, microcephaly, and abnormal gait (67).

We identified DDX3X as a candidate conveyer of OGT-CDG based on the presence of two highly conserved O-GlcNAc sites in the DDX3X C-terminal extension (CTE)—S584 and S588. The CTE is a highly conserved 26-residue stretch that is essential for DDX3X RNA unwinding activity (61). Deletion of this same region in the *Saccharomyces cerevisiae* ortholog Ded1p abolishes Ded1p trimerization (62), suggesting that the DDX3X CTE is required for association of DDX3X into functional trimers. Additionally, the CTE forms the majority of the region required for interaction with the mRNA export receptor TAP (residues 582–631) (68). The presence of two highly conserved O-GlcNAc sites in the CTE suggests that O-GlcNAcylation of DDX3X could modulate DDX3X trimerization and/or RNA unwinding activity. Alternatively, DDX3X O-GlcNAcylation could modulate nuclear mRNA export by altering the affinity of DDX3X for TAP. Both S584, S588, and the surrounding sequence are highly conserved in eukaryotes despite the region being intrinsically disordered (see File S1 for IUPred2A scores), further suggesting these residues are important for regulating DDX3X function.

### Hypo-O-GlcNAcylation of transcription factors may lead to transcriptomic dysregulation in OGT-CDG

**Figure 5:**
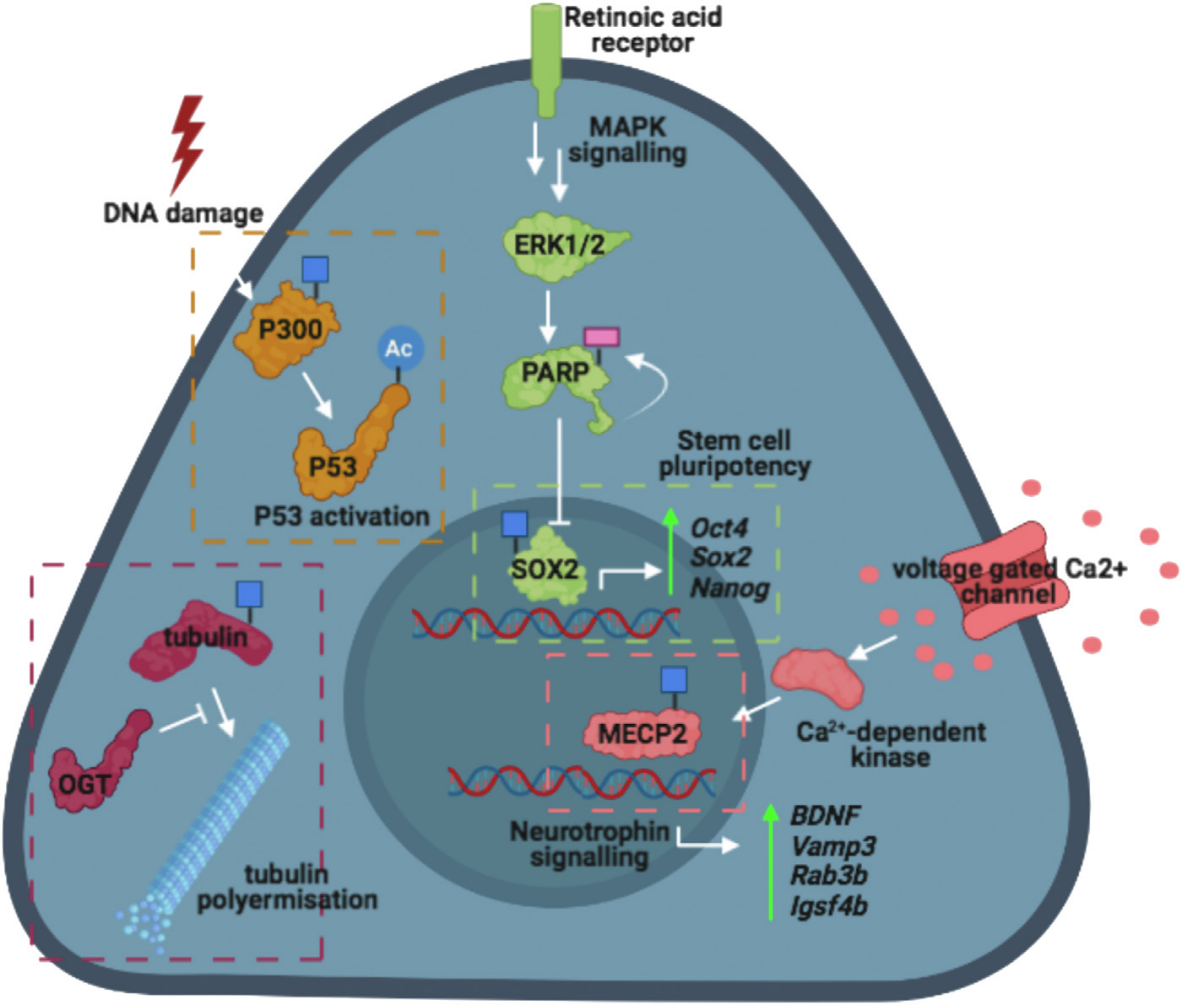
**Summary of the proposed functions of O-GlcNAc on selected examples of OGT-CDG candidate conveyers.** Hypothesized functions of O-GlcNAc on the OGT-CDG candidate conveyers are illustrated here. OGT-CDG candidate conveyers are annotated with a *blue square*, highlighting their O-GlcNAcylation status. Where expression of specific genes is expected to be affected by candidate O-GlcNAcylation status, these same genes are listed vertically in italics along with the expected change in expression following O-GlcNAcylation of the corresponding candidate protein. CDG, congenital disorder of glycosylation; OGT, O-GlcNAc transferase.

The link between O-GlcNAcylation and regulation of eukaryotic gene expression has been a source of considerable interest, with several components of the transcription/translation machinery being O-GlcNAc modified (43, 69, 70). For example, O-GlcNAcylation of RNA polymerase II and eIF4G modulates transcription and translation, respectively (43, 70). Of the candidates annotated as signaling nodes in neurogenesis (see GO analysis, Fig. 2*C*), the transcriptional regulator Methyl CpG binding protein 2 (MECP2), is a particularly interesting hit. MECP2 mutations are the predominant cause of Rett’s syndrome, an autistic spectrum disorder characterized by postnatal developmental regression, loss of motor skills, dystonia, loss of language/communication skills, and stereotypic hand movements (71). In resting neurons, MECP2 represses transcription at loci containing methylated CpG dinucleotides. This transcriptional repression is mediated *via* the scaffolding function of the MECP2 transcriptional repression domain, which recruits mSin3A, N-CoR, and c-Ski to silence transcription (72). Following neuronal depolarization, an unknown kinase phosphorylates MECP2 at S423 and S426, leading to dissociation of MECP2 from methylated loci and up-regulated expression of the neurotrophin *BDNF* (73). S426 is also a conserved site of O-GlcNAcylation on MECP2. Mice expressing a MECP2^S421A;S424A^ (S423 and S426 in human) knock-in display enhanced hippocampal-dependent learning and memory in a Morris water maze test and altered gene expression patterns (73). MECP2 O-GlcNAcylation has already been reported to be antagonistic to phosphorylation, with glucosamine supplementation elevating O-GlcNAcylation stoichiometry and reducing total MECP2 phosphorylation (32). However, the extent to which MECP2 S426 O-GlcNAcylation antagonizes neuronal depolarization-dependent changes in MECP2 phosphorylation and *BDNF* expression has yet to be investigated (See Fig. 5 for an illustrated summary of the previous discussion). Furthermore, it remains to be investigated whether hypo-O-GlcNAcylation of S426 in mature neurons dysregulates neuronal depolarization-dependent gene expression in the context of OGT-CDG.

In addition to the genetic landscape of differentiated neurons, disrupted gene expression patterns early in development could also contribute to OGT-CDG etiology. SOX2, a key pluripotency marker in embryonic stem cells (ESCs), was identified as a candidate conveyer of the OGT-CDG phenotype on account of the proximity of the O-GlcNAc site S246 to the SUMOylation site K247. SUMOylation of K247 reduces SOX2-dependent gene expression from the Fgf4 promoter due to reduced SOX2 DNA binding (74). Intriguingly, work by Myers *et al*.(75) found that mutagenesis of S246 to alanine in mESCs altered SOX2 genomic occupancy and increased SOX2 interaction with PARP1 (see later). Importantly, SOX2 loss-of-function variants give rise to an ID syndrome characterized by anophthalmia/microphthalmia, as well as developmental delay and increased risk of intracranial teratomas, indicating that hypo-O-GlcNAcylation of this essential pluripotency factor could contribute to OGT-CDG (76).

During retinoic acid–induced ESC differentiation, Erk1/2 phosphorylates PARP1 (77). This phosphorylation event stimulates auto-PARylation of PARP1 and promotes PARP1–SOX2 complex formation (78). In this complex, SOX2 is unable to bind Oct4-Sox enhancers and upregulate expression of key pluripotency genes including *Nanog*, *Oct4*, *Fgf4*, and *Sox2*, thus stimulating differentiation (78). These data are consistent with reports that SOX2 O-GlcNAcylation decreases with differentiation, and loss of O-GlcNAc on SOX2 increases PARP1 interaction, leading to reduced expression of genes required for maintenance of pluripotency (75). Whether PARP1–SOX2 complex formation is reduced during early stages of embryogenesis in OGT-CDG patients or in previously generated ESC models of the N567K and N648Y OGT mutations, has yet to be investigated. If complex stoichiometry is increased prematurely during embryogenesis, then premature ESC differentiation and dysregulated SOX2-dependent gene expression could contribute to the OGT-CDG phenotype.

In addition to transcription factors, a member of the histone acetyl transferase family of transcriptional coregulators—P300—was identified as a candidate conveyer of OGT-CDG in our analysis. P300 acetylates lysine residues on histones to facilitate chromatin unwinding and upregulate gene expression. During DNA damage, P300 is recruited to P53, where P300 upregulates P53-dependent transcription *via* histone acetylation and acetylation of five C-terminal lysine residues on P53 (79). The latter event activates the DNA-binding activity of P53 (79). P300 mutations are the second most common cause of Rubinstein-Taybi syndrome, which manifests in mild-to-moderate ID, growth delay, skeletal abnormalities, and facial dysmorphia (80). This study identified S1734 as a conserved O-GlcNAc site residing on the interface of the P300 Taz2 domain with the P53 N-terminal transactivation domain (TAD) (81). The P300 Taz2–P53 TAD interaction is predominantly mediated by hydrophobic contacts, though electrostatic and polar interactions between several arginine, glutamine, and serine residues also contribute to complex formation (81). S1734 forms hydrogen bonds to both T18 and Q17 of P53, stabilizing the complex. Phosphorylation of T18 on P53 increases the affinity of the Taz2–TAD complex 11-fold, indicating this residue is particularly important for modulating the affinity of P53 for P300 (81). The presence of a bulky GlcNAc moiety could be hypothesized to act antagonistically to T18 phosphorylation, by inducing a steric clash between S1734 and T18/Q17 on the Taz2–TAD interface. This in turn would likely exert negative downstream effects on P53-dependent transcriptional activation due to reduced P300 recruitment and subsequent reductions in the levels of histone acetylation. Overall, P300 O-GlcNAcylation at S1734 may negatively regulate P300–P53 complex formation, as indicated by prior structural studies showing a close proximity of the S1734 hydroxyl group to the regulatory P53 phospho-site T18 and Q17 (See Figure 5 for an illustrated summary of the previous discussion). Hypo-O-GlcNAcylation of this site in the context of OGT-CDG could potentially lead to dysregulated P53 activity, which is essential for cell cycle arrest and apoptosis in response to DNA damage. To investigate the functional effects of O-GlcNAc on P300, *in vitro* acetylation of P53 by S1734 O-GlcNAcylated P300 and GlcNAc-deficient P300 could be analyzed as described previously (79).

### Identification of pathogenic missense variants at O-GlcNAcylation sites

So far, this study has focused on predicting functional O-GlcNAc sites on ID/DD-associated proteins using the filter-based approach outlined in Figure 1. Because mutations in these proteins segregate with ID/DD, it is also possible that hypo-O-GlcNAcylation (*i.e.*, mutations of Ser/Thr to other amino acids) of functional O-GlcNAc sites on these proteins may contribute to OGT-CDG pathology. We explored this possibility across the entire O-GlcNAcome (*i.e.*, not limited to the candidates in Fig. 1).

To identify mutations at O-GlcNAc sites that segregate with ID/DD, the ClinVar database (82) was screened for missense variants using the O-GlcNAc database (83), a recently compiled database of O-GlcNAc sites reported across multiple eukaryotic species. We also screened for missense variants ±5 residues from the O-GlcNAc site, as mutations proximal to the O-GlcNAc site may affect the affinity of OGT for a given substrate. The ClinVar screen identified 125 missense variants on O-GlcNAc sites, eight of which segregated with ID/DD (See File S2 for the list of ClinVar hits). The remaining 117 O-GlcNAc site missense variants were either pathogenic for a non-ID/DD syndrome or scored as likely benign or possibly damaging by PolyPhen scoring (PolyPhen HumVar score < 0.85) Additionally, we identified 34 pathogenic and likely pathogenic variants proximal (±5 residues) to the O-GlcNAc site, which segregate with ID/DD (See File S2). Two O-GlcNAcylation site mutations (TUBB2B^S172P^ and TUBB2B^S172L^) were pathogenic or likely pathogenic in patients with cortical dysplasia, complex, with other brain malformations (CDCBM7) (49). Six O-GlcNAcylation site missense variants (PUM1^S802F^, DOCK7^S190N^, DOCK7^S190G^, KMT2A^S1858I^, ATRX^S594C^, and HCF1^T556M^) were of uncertain significance, although PolyPhen scoring predicted all six variants as probably pathogenic (PolyPhen HumVar score > 0.85). For summaries of ID/DD missense variants and associated pathogenicity/PolyPhen scores, see File S2.

Of the hits identified by the ClinVar search, the pathogenic TUBB2B^S172P^ mutation was particularly interesting. The TUBB2B^S172P^ variant segregates with CDCBM7 and displays impaired heterodimerization with α-tubulin and is not capable of polymerizing into microtubules (49). A similar effect would be predicted from the presence of a bulky O-GlcNAc moiety at S172 due to steric occlusion of the GTP/GDP-binding site (see earlier discussion and Fig. 3*A*), further substantiating the proposed role of this site in regulating tubulin polymerization. Additionally, an S172L variant was also reported for the CDCBM7 syndrome (Table 1); however, the effects of this mutation on tubulin function have not been investigated biochemically or structurally. It is of course possible that these mutations affect tubulin function independent of O-GlcNAcylation. Indeed, mutagenesis from Ser to Pro (a “helix breaker” with restricted backbone conformations) may disrupt tubulin function independently of the loss of O-GlcNAc. However, the backbone conformation of S172 is compatible with that preferred by a proline (Fig. 3*B*). Future work could explore the effects of proline mutagenesis and loss of S172 O-GlcNAcylation on tubulin function in ID/DD.

From the six missense variants predicted to be pathogenic ID/DD mutations by PolyPhen scoring, the HCF1^T566M^ variant is of particular interest. The HCF1^T566M^ variant is present in a patient with mental retardation 3, X-linked. T566 was not identified as a candidate O-GlcNAc site in our initial filter-based approach as it is not in a (known) functional region/domain, although it is proximal to the binding site for OGT (84). It is possible, however, that T566 O-GlcNAcylation stabilizes HCF1; HCF1 is cotranslationally O-GlcNAcylated (85), and the sole reported function of cotranslational O-GlcNAcylation is stabilization of nascent protein chains during translation (39). Thus, the HCF1^T566M^ variant may represent a destabilized hypomorph due to loss of T566 O-GlcNAcylation. Whether this is the case and whether HCF1 hypo-O-GlcNAcylation at T566 contributes to OGT-CDG etiology, are avenues for future research.

## Conclusions

We have identified 22 ID/DD-associated proteins, the hypo-O-GlcNAcylation of which may contribute to OGT-CDG pathology. We arrived at these candidates by inferring functionality for conserved O-GlcNAc sites on these ID/DD-associated proteins through a filter-based approach integrating inputs from proteomic, clinical, structural, and literature databases. Additionally, we identified several conserved O-GlcNAc sites, which are pathogenically mutated in syndromic ID patients, adding further impetus to study the function of O-GlcNAc on these neuronal proteins. We anticipate that the results of this study will further highlight the convergence between the O-GlcNAc proteome and ID and galvanize further research into specific O-GlcNAc signaling nodes and how hypo-O-GlcNAcylation of these same proteins may give rise to OGT-CDG.

## Experimental procedures

### Filter-based approach for the identification of OGT-CDG candidate conveyers

O-GlcNAc proteomic (O-GlcNAcomic) data were pooled manually from 14 publications into a Microsoft Excel spreadsheet (version 16.46) (9, 10, 11, 25, 26, 29, 30, 31, 32, 33, 34, 86, 87, 88). As a quality control step, O-GlcNAc sites were excluded if the reported O-GlcNAc site mapped to asparagine (N-linked glycosylation), if the corresponding peptide was misannotated (reported O-GlcNAcylated peptide absent from the UniProt FASTA sequence of the full-length protein) or if the protein was exclusively extracellular (determined by manual inspection of UniProt entries for protein localization). To assess whether an O-GlcNAcylated protein was ID/DD associated, the list of site-mapped O-GlcNAcylated proteins was then compared to a recently reported list of all known ID/DD-associated genes (22) and to the DECIPHER database of clinical genomic variation (35). If the gene encoding an O-GlcNAcylated protein was reported as being the sole “pathogenic” variant in a patient with “ID” or “developmental delay,” the O-GlcNAcylated protein passed this filter. Subsequently, to assess whether O-GlcNAc sites were conserved in vertebrates, amino acid sequences in FASTA format for human, mouse, rat, rabbit, and sheep homologs of a given O-GlcNAcylated protein were extracted from the UniProt database (see File S1 for UniProt IDs used). Clustal Omega was used to align amino acid sequences, with O-GlcNAc sites not conserved in ≥1 species excluded. In 3.84% of cases, one of rat, rabbit, or sheep sequences were absent for a given protein. In such cases, bovine, porcine, or chimpanzee sequences (in descending order of preference) were used instead, due to their availability in the UniProt database. Structural inspection of O-GlcNAc sites, to assess whether O-GlcNAc sites were surface exposed and thus accessible to the OGT active site, was carried out on available PDB structures using PyMOL (https://pymol.org/2/) v 1.7.4.5. Where structural information was not available, the IUPred2A (https://iupred2a.elte.hu) disorder predictor was used to predict whether an O-GlcNAc site resided in a disordered (and therefore accessible) region of the protein, using the long disorder (default) setting (40). O-GlcNAc sites were classified as ordered if IUPred scores were higher than 0.5. Disorder predictions were carried out using FASTA sequences from human homologs. IUPred scores for individual O-GlcNAc sites are detailed in File S1.

### GO and PANTHER pathway analysis

GO analysis for biological processes involving OGT-CDG candidate conveyers was carried out using the Gene Ontology resource (http://geneontology.org) (89). For investigating pathways in which OGT-CDG candidate conveyers are signaling nodes and classifying candidate proteins into classes, PANTHER version 16.0 was used (90). For both GO and PANTHER pathway analysis, UniProt identifiers for human homologs were used (see File S1). Outputs from classification of shared “biological processes,” “protein class,” and “pathways” were exported as .txt files into Microsoft Excel and used to generate the associated figures.

### ClinVar database screening for missense mutations in ID/DD-associated proteins

To retrieve variants in genes encoding O-GlcNAc modified proteins, the ClinVar short variants dataset (82) (https://ftp.ncbi.nlm.nih.gov/pub/clinvar/vcf_GRCh38/clinvar.vcf.gz, accessed 03/01/2022) was parsed using the PyVCF library and was cross compared with a recently published O-GlcNAcome (83) and an independent list of 146 conserved O-GlcNAc sites (File S1) parsed using the csv library (91). Identified variants were filtered to only include missense mutations. Amino acid substitutions for each variant were retrieved using the requests library (92) to interface with the esummary ClinVar API, parsed using the json library. Missense mutations affecting O-GlcNAcylated residues or sites ±5 residues were then filtered based on pathogenicity and retained for manual follow up. Code can be accessed at https://github.com/IggyCz/ClinVar_O-GlcNAcome_search/blob/main/clinvar_searcher_v1.py.

### PolyPhen scoring

PolyPhen v2 (93) was used to predict the pathogenicity of missense variants at conserved sites of O-GlcNAcylation. UniProt FASTA sequences for human homologs of a given candidate conveyer were manually entered into the search bar. HumVar scores, which factor in the presence of single nucleotide polymorphisms at a given site in healthy individuals, were used, along with default cut-offs of 0.85 for “probably damaging” and 0.15 for “likely benign.”

## Data availability

All data are contained within the article.

## Supporting information

This article contains supporting information (14, 49, 50, 56, 59, 60, 61, 68, 72, 74, 75, 81, 84, 94, 95, 96, 97, 98, 99, 100, 101, 102, 103, 104, 105, 106, 107, 108, 109, 110).

## Conflict of interest

The authors declare that they have no conflicts of interest with the contents of this article.
